# Arabidopsis *weep* mutants exhibit narrow root angles

**DOI:** 10.17912/micropub.biology.000584

**Published:** 2022-06-06

**Authors:** Joy M Johnson, Andrea R Kohler, Miranda J Haus, Courtney A Hollender

**Affiliations:** 1 Michigan State University Department of Horticulture

## Abstract

Peach ( *Prunus persica* ) trees with a mutation in the *weep* gene exhibit a weeping branch phenotype. In contrast, Arabidopsis ( *Arabidopsis thaliana* ) *weep* mutants do not have a shoot architecture phenotype. A recent report revealed that barley ( *Hordeum vulgare* ) and wheat ( *Triticum aestivum* ) with mutations in EGT2, a *WEEP* homolog, have steeper root angles than standard varieties. We investigated the root architecture of three Arabidopsis *weep* mutant lines. All three lines exhibited steeper root angles and a smaller convex hull area, indicating that the total area explored by the root system is reduced. These results reveal *WEEP* is important for regulating lateral root angles in a dicot.

**Figure 1. Arabidopsis weep mutants exhibit steeper lateral root angles than control plants:**
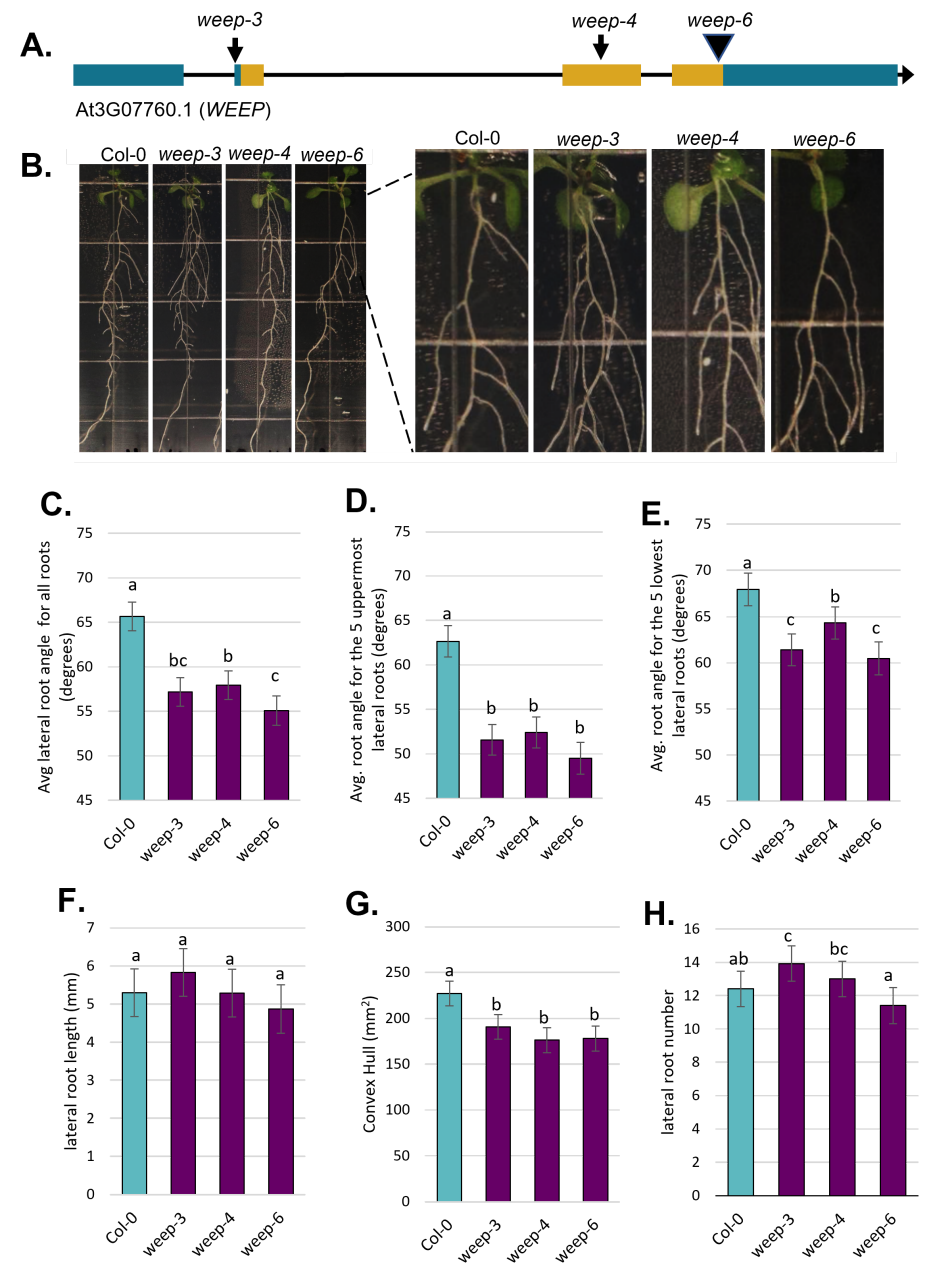
(A) Cartoon illustrating the *WEEP* gene structure for the At3G07760.1 splice form, along with the relative locations of the *weep-3* , *weep-4* , and *weep-6* mutations. Blue coloring indicates UTRs and yellow indicates coding sequence regions. (B) Representative images of 9-day old control (Col-0), *weep-3* , *weep-4* , and *weep-6* plants on agar plate containing ½ MS + 1% sucrose. Images were taken from the same experiment. Images on right are enlargements of the upper two boxes of the plate. (C) Average lateral root angle for the four genotypes. (D) Average lateral root angle for the five oldest (uppermost) roots. (E) Average lateral root angle for the five youngest (lowest) roots. (F) Average root length of lateral roots initiating 1 to 2 cm below the shoot. (G) Average convex hull and (H) average lateral root number for the Col-0, *weep-3* , *weep-4* , and *weep-6* plants. Error bars in C –H represent standard error of the mean and bars with the same letter are not significantly different at α=0.05 in all pairwise comparisons.

## Description

The *WEEP* gene codes for a highly conserved protein found throughout vascular plants and some mosses (Hollender *et al,* 2018). *WEEP* is expressed broadly across plant organs, including in shoots, leaves, fruits, and roots (Kee et al. 2009; Denay et al. 2017; Hollender et al. 2018; Kirschner et al. 2021). The only known functional domain in WEEP is a Sterile Alpha Motif (SAM), a domain which commonly functions as a binding site, allowing protein-protein, protein-RNA, or protein-lipid interactions (Qiao and Bowie, 2005; Denay *et al* ., 2017; Hollender *et al.* , 2018; Kirschner et al., 2021). Despite high sequence conservation, *WEEP* has been reported to play diverse roles in different species, and little is known about its function. The *Cucumis melo* (melon) homolog, named *DOWNWARD LEAF CURL (CmDLC* ), appears to play a role in control of cell size, as it is highly expressed in enlarging fruit, and *CmDLC* overexpression in Arabidopsis resulted in leaf epinasty due to decreased leaf epidermal cell size and number, with a larger decrease on the abaxial side than the adaxial (Kee et al. 2009). In *Prunus persica* (Peach) *WEEP* is an important regulator of branch orientation (Hollender et al. 2018). Homozygous peach *weep* mutants have pendulous branches which follow an elliptical downward trajectory, rather than a linear upward trajectory as in standard peach (Hollender et al. 2018). Similarly, RNAi-mediated silencing of *WEEP* in plum ( *P. domestica* ) led to wandering and arching shoot orientations (Hollender et al. 2018). Arabidopsis T-DNA plants with reduced expression of the *WEEP* homolog At3g07760 (Salk_135361; *weep-5* ) did not exhibit abnormal shoot phenotypes, nor did four CRISPR/Cas9- generated null mutants (Kee et al. 2009; Hollender et al. 2018). However, some genes and gene families that control shoot architecture traits, such as branch angle, also influence root architecture (Tworkoski and Scorza 2001; Gaudin et al. 2014; Taniguchi et al. 2017; Waite and Dardick 2021). Recently, the *WEEP* homologs in barley ( *Hordeum vulgare* ) and wheat ( *Triticum aestivum* ), named *ENHANCED* *GRAVITROPISM2* ( *EGT2* ), were determined to be involved in root architecture and gravitropic response (Kirschner et al. 2021). In these species, *egt2* mutants exhibited narrower seminal and lateral root angles and a hyper-gravitropic response, but normal root number and length (Kirschner et al. 2021).

Root architecture significantly impacts nutrient and water uptake. Accordingly, understanding the molecular and genetic factors that regulate root architecture can inform breeding programs for both horticultural and cereal crops. Here, we examined the root architecture of three Arabidopsis *weep* mutant lines: *weep-* 3 and *weep-4* (from Hollender *et al.,* 2018), and a previously undescribed line with a T-DNA insertion in the end of the *WEEP* locus (SALK_052005), which is now identified as *weep-6* (Figure 1A). The *weep-4* and *weep-6* mutations disrupt coding sequence in all eleven reported splice forms, while the *weep-3* mutation disrupts the coding sequence in four (At3G07760.1 , At3G07760.2, At4G07760.5, and At4G07760.10). Consistent with the phenotype reported by Kirschner *et al.* in barley and wheat, all three Arabidopsis *weep* mutant lines exhibited steeper (narrower) lateral root angles when compared to the wild type control (Columbia Col-0; Figure 1B-E) (Kirschner et al. 2021). Lateral root angle differences were most pronounced when comparing the oldest (upper) roots, closest to the shoot (Figure 1D). However, the youngest (lower) lateral roots on the *weep* mutants were still steeper than Col-0 (Figure 1E). In addition, mean lateral root lengths did not differ between genotypes (Figure 1C). The roots of all genotypes had reached the bottom of their vertically oriented agar plates when measurements were taken. Thus, there were no differences in primary root length. Accordingly, the steeper lateral root angles in the *weep* mutants alone led to them having a smaller convex hull, which reflects the area occupied by the root system (see methods for details) (Figure 1G). Lastly, lateral root number did not appear to be impacted by the presence of *weep* mutations (Figure 1H).

These observations suggest *WEEP* promotes shallower lateral root growth via narrower root angles in Arabidopsis, and likely other dicots due to its high conservation at the protein level. Shallower root angles are beneficial in low phosphorous situations (Lynch 2011; Li et al. 2016). On the other hand, steeper root angles are agronomically valuable because they can provide plants with better access to water and nutrients deep in the soil, particularly nitrogen, as well as provide greater anchorage (Paez-Garcia et al. 2015; Li et al. 2016). Manipulating *WEEP* expression could therefore provide a method of controlling the shape of root architecture in many crop species. This could be particularly useful for rootstock for crops that are vegetatively propagated through grafting. Experiments in peach indicated that *WEEP* acts locally and does not produce a systemic signal, suggesting rootstock with modified *WEEP* expression could achieve the improved root architecture without causing pleiotropic effects in scion (Hollender et al. 2018) . Further studies are needed to investigate the molecular mechanism behind lateral root control by *WEEP* , as well as the utility of modifying *WEEP* expression in crop plants.

## Methods

***Plant Germplasm and Growth Conditions***

The Arabidopsis germplasm used for this study was *Arabidopsis thaliana* Columbia-0 (Col-0) for the control, T-DNA line SALK_052005 ( *weep-6* ), and two previously described CRISPR-generated lines *weep-3* and *weep-4* (Hollender et al. 2018). All mutant lines are in the Col-0 background. Arabidopsis *weep-3* plants contain several indels ( *in* sertions or *del* etions) upstream and just past the At3G07760.1 start codon, *weep-4* plants contain a single insertion of an ‘A’ in the second exon (948_949insA), and *weep-6* contains a large insertion in the last (3 ^rd^ ) exon, impacting a putative phosphorylation site. Seeds were surface sterilized by shaking in 70% ethanol with 0.5% triton for five minutes followed by soaking for 15-minutes in 95% ethanol before being air dried. Four seeds were sown at equal spacing along the outermost line of a gridded square petri dish (plate) containing 1/2 MS media with 1% sucrose and 0.8% agarose. Plates were sealed with micropore tape, placed in the dark at 4°C for three days for seed stratification, and then vertically oriented on a growth rack in a 21°C room and left to grow under 24-hour light (~ 120 uMolm ^-2^ s ^-1^ ). Five plates per genotype were grown for all four genotypes simultaneously. This entire experiment was repeated three times.

***Phenotyping***

After 9 days under light, photographs of each plate were captured using a Cannon EOS M5 Digital SLR. Image J ( https://imagej.nih.gov/ij/ ) was used to manually measure lateral root angles, lateral root length, and convex hull from the photographs. Lateral root emergence angles were manually measured for every lateral root on each plant using the angle measurement tool by tracing a line straight through the primary root, making a point at each root node and then tracing the initial trajectory of the lateral root. Lateral root length measurements were taken from the roots that initiated between 1 and 2 cm below each shoot (in the second box below the cotyledons). Convex hull was measured manually using the "polygon selections" function in ImageJ to get a 2-dimensional area measurement according to the following method: the first point for each plant measurement was placed directly below the shoot, additional points were manually added in a counterclockwise manner to outline the widest parts of the roots, and the last point was the same as the first.

***Statistical Analysis***

Statistical analysis of root phenotype data was performed in R (v. 4.1.2) using the R package "lme4". Multiple comparison testing was performed using the packages "emmeans," "multcomp," "lmerTest." Type 3 ANOVAs were performed using "car”. Root angle, root length, root number, and convex hull (response variables) were modeled as a function of genotype, blocked by experimental date. Each plate was treated as an experimental unit, with plants (and individual roots, if applicable) treated as subsamples. The final model was (Response Variable) ~(Mean) + (Genotype) + (Block) + (Plate: Block:Genotype) + (Plant:Plate: Block:Genotype) + (Error), where (Genotype) is the fixed effect of each genotype, (Block) is the random effect of experimental block, (Plate: Block:Genotype) is the random effect of plate (nested in Block by Genotype interaction), and (Plant:Plate: Block:Genotype) is the random effect of plant (nested in Plate). For root number and convex hull, where there is only one measurement per plant, the term for the random effect of plant was not used. For root angle by position, (Position)—the fixed effect of the lateral root’s location on the primary root—and (Position*Genotype)—the interaction effect of Position and Genotype—were added to the model. Residuals of each model were checked to determine whether the data was normal with equal variances. Each model was gated by type 3 ANOVA (α=0.05). If the ANOVA showed a significant effect of Genotype, all pairwise comparisons were performed using t-tests (α=0.05). Otherwise, all pairwise comparisons were performed using Šidák correction (α=0.05). For root angle by position, genotype and position interaction was significant, so pairwise comparisons were performed slicing by position.
